# Hydrothermal Synthesis of Nanooctahedra MnFe_2_O_4_ onto the Wood Surface with Soft Magnetism, Fire Resistance and Electromagnetic Wave Absorption

**DOI:** 10.3390/nano7060118

**Published:** 2017-05-23

**Authors:** Hanwei Wang, Qiufang Yao, Chao Wang, Zhongqing Ma, Qingfeng Sun, Bitao Fan, Chunde Jin, Yipeng Chen

**Affiliations:** 1 School of Engineering, Zhejiang Agriculture & Forestry University, Lin’an 311300, China; 18868196590@163.com (H.W.); yaoqiufang105@163.com (Q.Y.); chaowangzafu@163.com (C.W.); mazqzafu@163.com (Z.M.); 18357178962@163.com (B.F.); jincd@zafu.edu.cn (C.J.); 18868195633@sina.cn (Y.C.); 2 Key Laboratory of Wood Science and Technology, Hangzhou 311300, China

**Keywords:** wood, MnFe_2_O_4_/wood composite, hydrothermal, soft magnetism, fire resistance, electromagnetic wave absorption

## Abstract

In this study, nanooctahedra MnFe_2_O_4_ were successfully deposited on a wood surface via a low hydrothermal treatment by hydrogen bonding interactions. As-prepared MnFe_2_O_4_/wood composite (MW) had superior performance of soft magnetism, fire resistance and electromagnetic wave absorption. Among them, small hysteresis loops and low coercivity (<±5 Oe) were observed in the magnetization-field curve of MW with saturation magnetization of 28.24 emu/g, indicating its excellent soft magnetism. The MW also exhibited a good fire-resistant property due to its initial burning time at 20 s; while only 6 s for the untreated wood (UW) in combustion experiments. Additionally, this composite revealed good electromagnetic wave absorption with a minimum reflection loss of −9.3 dB at 16.48 GHz. Therefore, the MW has great potential in the fields of special decoration and indoor electromagnetic wave absorbers.

## 1. Introduction

Wood/inorganic hybrids fall under the category of new functional nanocomposite materials because of being a combination between organic and inorganic materials with their respective advantages and excellent performance. Synergistic effects resulting from the physical or chemical interactions between the inorganic and wood components have produced such properties as improved thermal stability, UV-resistance, hydrophobic, mechanical and dimensional stability [1]. Currently, deposition of a thin solid inorganic nanomaterial coating imbedded onto the wood surface has great potential to improve the inherent wood defects (like moisture deformation, easily burnt, etc.) and simultaneously to grant novel performances (like super-hydrophobic, self-cleaning, UV–resistance, or others) [2]. The present focus among the thin solid inorganic nanomaterial coatings is mainly metal semiconductor materials, like TiO_2_ [3,4], SiO_2_ [5], ZnO [6], CeO_2_ [7], and so on [8,9,10,11,12,13]. Several research works paid less attention to magnetic nanostructure materials deposited onto the wood surface.

With the rapid development of wireless communications indoors and outdoors, the electromagnetic interference (EMI) pollution has become much more serious. The electromagnetic waves may cause interception and malfunction of the performance of electrical equipment in medical, military and aircraft systems or even lead to radiative damage of the human body [7,14]. Therefore, it is necessary to exploit new types of microwave-absorption materials with excellent properties, such as a wide frequency range, strong absorption, low density, high resistivity, etc. 

Magnetic nanomaterials/wood hybrids would be potential candidates for microwave absorption, especially when wood serves as interior decorations due to its renewability, attractive surface, sound insulation, temperature- and humidity-controlling performances. It may be a reasonable choice for wave absorption if the wood surface is embedded with a thin solid magnetic film with a trivial change of appearance. Previous studies have been conducted showing that the wood surface can be considered as an effective substrate containing plentiful hydroxyl groups for the nucleation and growth of inorganic nanomaterials. Publicly reported pathways for the deposition of magnetic materials are the sol-gel method [15,16,17], electroless deposition [18], the hydrothermal process [19,20] and physical padding. Among these methods, the hydrothermal method was a feasible and efficient pathway for growing magnetic nanomaterials with high product purity and homogeneity, crystal symmetry, narrow particle size distributions, a lower sintering temperature, a wide range of chemical compositions and single-step processes, as well as for the growth of crystals with polymorphic modifications [21,22,23,24,25,26,27]. Herein, we employed a facile low temperature hydrothermal process for the growth of nanooctahedra MnFe_2_O_4_ on the wood surface. The as-prepared MnFe_2_O_4_/wood (MW) composite showed a superior soft magnetism, fire resistance and electromagnetic absorption. The saturation magnetization of the MW was 28.24 emu/g with extremely small hysteresis loops, and low coercivity indicated that this composite was an excellent soft-magnetic material. The MW also exhibited a good fire resistance property due to it not being burnt in the first 20 s, while only 6 s were needed for the untreated wood. Additionally, this composite is a good electromagnetic absorption material due to its minimum reflection loss of −9.3 dB at 16.48 GHz. Thus, the MW has great potential in the fields of special decoration and indoor electromagnetic wave absorbers.

## 2. Experimental Details

### 2.1. Materials

All chemicals were supplied by Boyle chemical Co. Ltd., Shanghai, China. and used without further purification. The wood slices were cut with sizes of 20 mm (length) × 10 mm (width) × 5 mm (height), and then, the slices were ultrasonically rinsed in deionized water for 30 min and dried at 80 °C in a vacuum.

### 2.2. One-Pot Hydrothermal Synthesis of MW 

In a typical synthesis, FeCl_3_·6H_2_O and MnSO_4_·H_2_O in a stoichiometric ratio of 2:1 were dissolved in 80 mL of deionized water under magnetic stirring at room temperature. The obtained homogeneous mixture was transferred into a 100 mL Teflon-lined stainless autoclave. Wood specimens were subsequently placed into the above reaction solution, and the pH value was adjusted via adding a certain amount of ammonia solution. The Teflon-lined stainless-steel autoclave was sealed and heated to 120 °C for 8 h. Subsequently, the autoclave was left to cool down to room temperature. Finally, the prepared magnetic wood samples were removed from the solution, ultrasonically rinsed with deionized water for 30 min and dried at 45 °C for over 24 h in a vacuum. 

### 2.3. Characterizations

The surface morphologies of the samples were characterized by scanning electron microscopy (SEM, Quanta 200, FEI, Eindhoven, The Netherlands). Crystalline structures of the samples were identified by the X-ray diffraction technique (XRD, D/MAX 2200, Rigaku, Tokyo, Japan) operating with Cu Kα radiation (λ = 1.5418 Å) at a scan rate (2θ) of 4° min^−1^, an accelerating voltage of 40 kV and the applied current of 30 mA ranging from 10–80°. Changes of chemical groups were recorded via a Fourier transform infrared spectroscopy (FT-IR, Magna-IR 560, Nicolet, Madison, WI, USA). XPS analysis was characterized by an X-ray photoelectron spectrometer (XPS, ESCALAB 250 XI, Thermofisher Co., Bridgewater, NJ, USA). The magnetic properties of the composites were measured by a vibrating sample magnetometer (VSM, Model 7404, LakeShore Cryotronics Inc., Westerville, OH, USA) at 300 K. The thermal performances of the MW were examined using a thermal gravimetric analysis (TGA, SDT Q600, TA Instruments, New Castle, DE, USA) heating rate of 4 °C/min and under an N_2_ flow rate of 50 mL/min. The relative permeability and permittivity were obtained on an Agilent N5244A PNA-X network analyzer (VNA, Agilent Technologies Inc., Richardson, TX, USA) in the frequency range of 2–18 GHz for the calculation of reflection loss (RL) by the coaxial reflection/transmission method based on the NRW (Nicolson-Ross-Weir) method. The sample containing composite materials and paraffin wax with the mass ratio of 2:3 was pressed into toroidal-shaped samples (Φout = 7.00 mm, Φin = 3.04 mm, thickness = 2 mm) for microwave measurement. The simulated reflection loss (RL) was calculated from the measured parameters according to the transmission line theory. 

## 3. Results and Discussion

**Figure 1:**
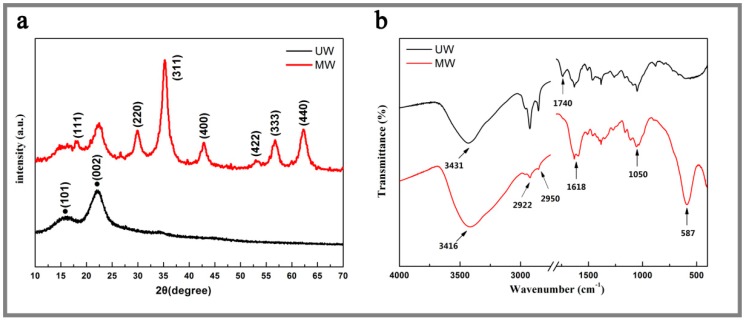
(**a**) XRD patterns and (**b**) FTIR spectra of the untreated wood (UW) and the MnFe_2_O_4_/wood composite (MW).

Figure 1a shows the XRD patterns of the untreated wood and the MnFe_2_O_4_/wood composite. The strong diffraction peaks at 16.1° and 22.6° were equivalent to the crystalline region of the cellulose of the wood (Figure 1a). In addition, the diffraction peaks at 17.9°, 30.3°, 35.6°, 43.3°, 53.5°, 57.1° and 62.8° could be attributed to the diffractions of the (111), (220), (311), (400), (422), (511) and (440), which confirmed the presence of the MnFe_2_O_4_ (JCPDS 73-1964) with a phase-pure spinel structure on the wood surface. This result revealed that the MnFe_2_O_4_ was successfully grown on the wood surface.

Figure 1b showed the FTIR spectra (400–4000 cm^−1^) of the untreated wood (UW) and the MW. The main absorption bands of the MW were located at 3416 cm^−1^, 1168 cm^−1^ and 1050 cm^−1^, corresponding to the stretching vibrations of O–H, C=O and C–O, respectively, which were attributed to the chemical contents of the wood substrate (hemicellulose, cellulose and lignin). The peak of UW at 3431 cm^−1^ was ascribed to stretching vibrations of the hydroxyl groups of the wood. Then, this peak shifted to the lower wavenumbers of 3416 cm^−1^ after coating MnFe_2_O_4_ on the wood surface, indicating the formation of the hydrogen bonding interaction between the nanooctahedra MnFe_2_O_4_ and the wood [28]. The peaks at 2922 cm^−1^ and 2950 cm^−1^ were caused by the stretching vibrations of –CH_3_ and –CH_2_, but these peaks in the MW spectra significantly decreased; and the peak at 1740 cm^−1^ (the carbon and oxygen double bonds) only existed in the UW spectra [29]. These phenomena were attributed to the hydrolysis of fatty acids by hydrothermal reaction under the alkaline conditions. Simultaneously, the absorption peaks at 587 cm^−1^ were intrinsic vibrations of the manganese ferrite [29].

**Figure 2:**
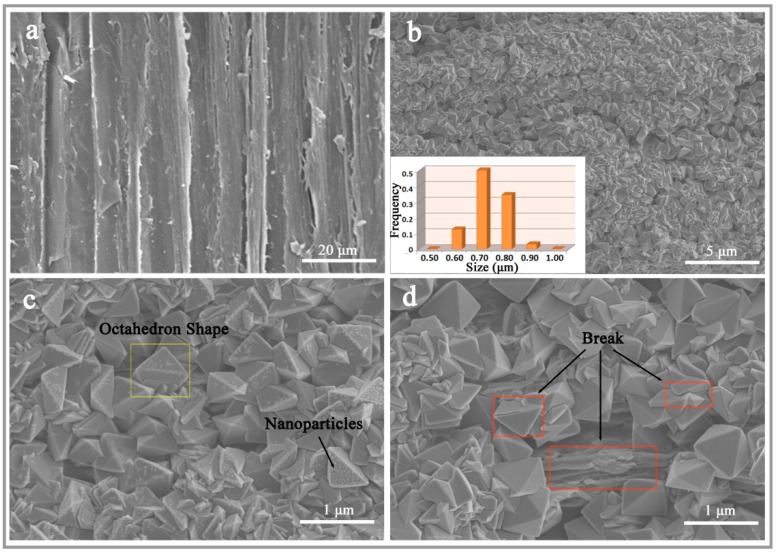
SEM images of (**a**) the surfaces of the UW; (**b**) the surfaces of the MW with low-magnification; (**c**) the surfaces of the MW without ultrasonic treatment and (**d**) the surfaces of the MW after ultrasonic treatment. The inset of (**b**) is the size distribution of nanooctahedra MnFe_2_O_4_.

The surface morphologies of the UW and MW by scanning electron microscopy are exhibited in Figure 2. Some fibrils and the inner surface of the lumen could be clearly observed on the microstructure of a longitudinal section of the UW surface. After the hydrothermal process, the wood surface was compactly covered by MnFe_2_O_4_ in Figure 2b. The inset of (b) shows the size distribution of nanooctahedra MnFe_2_O_4_ with an average particle size of 0.68 nm and a size distribution width of 0.5–1 μm. Figure 2c shows the MnFe_2_O_4_ with an octahedral shape aggregated over the wood surface by a compact manner, and many small nanoparticles also were equipped on this nanooctahedra MnFe_2_O_4_ surface. In order to further investigate the fine features of the nanooctahedra MnFe_2_O_4_, the MW was studied by a zoomed-in SEM after ultrasonic treatment with 1800 W for 30 min (Figure 2d). The zoomed-in SEM showed the nanooctahedra MnFe_2_O_4_ still tightly covered the wood surface, but a small fraction of the MnFe_2_O_4_ and the bare wood surface appeared to rupture. These results indicated that the nanooctahedra MnFe_2_O_4_ were closely integrated with the wood surface by a strong interaction. In addition, the surface of nanooctahedra MnFe_2_O_4_ became very clean, which might reveal that small nanoparticles were adsorbed on the surface of nanooctahedra MnFe_2_O_4_ by a physical process.

**Figure 3:**
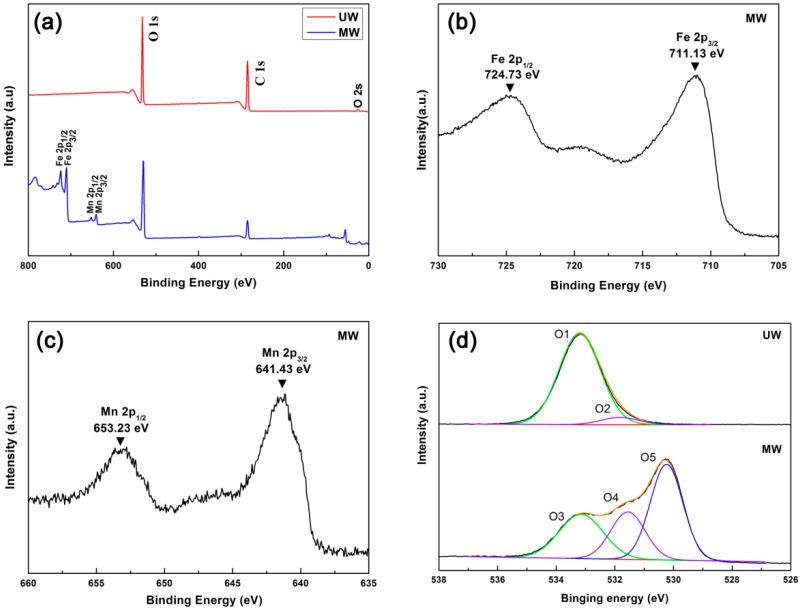
(**a**) Survey-scan XPS spectra of the UW and the MW; (**b**) Fe2p XPS spectra of the MW; (**c**) Mn2P XPS spectra of the MW; (**d**) O1s XPS spectra of the UW and MW.

X-ray photoelectron spectroscopy (XPS) is a powerful technique for studying the elemental composition and chemical oxidation state of the surfaces. Figure 3a shows the survey-scan XPS spectra of UW and MW; elemental O and C coexisted in both samples, where the C element was attributed to the wood substrate from cellulose, lignin and hemicellulose. In addition, besides O and C elements, the survey-scan spectrum of the MW revealed iron and manganese peaks, which should come from MnFe_2_O_4_ nanoparticles on the wood surface. Figure 3b,c shows the Fe2p and the Mn2p spectra, respectively. For Fe, the two main peaks Fe2p_1/2_ and Fe2p_3/2_ at 724.73 eV and 711.13 eV, respectively, indicate the presence of Fe^3+^ cations by comparison with the Fe2p_3/2_ peaks of Fe_2_O_3_ and Fe metal [30,31,32,33]. Two sharp peaks for the Mn2p_1/2_ and Mn2p_3/2_ at 653.23 eV and 641.43 eV, respectively, indicate the presence of Mn^2+^ cations by comparison with the MnO [34].

Figure 3d and Table 1 illustrated the O1s spectra and binding energy of the UW and MW samples, respectively. The broad peak of O1s at the UW spectrum could be fitted by two peaks at binding energies of 533.18 eV and 531.83 eV, respectively. The largest peak at 533.18 eV was attributed to the carbon-oxygen bond on the wood, such as cellulose, lignin and hemicellulose. The other peak at 531.83 eV was assigned to the other oxygen from OH, H_2_O and carbon-oxygen double bonds from the wood substrate. By contrasts, the main peak on the O1s spectrum of MW appeared at binding energies of 530.23 eV, which was compatible with oxygen in metal oxides, such as Fe–O and Mn–O [35,36]. Furthermore, it is easily observed that the peak at 531.82 eV had been shifted to 531.55 eV and relatively higher than before the reaction. That strongly indicated that the MnFe_2_O_4_ nanoparticles had been successfully located on the wood surface by the hydrogen bond after hydrothermal treatment under this work.

A mechanism of the formation of the nanooctahedra MnFe_2_O_4_ might be expressed by reaction Equations (1)–(3):

Fe^3+^ + 3OH^–^⇋Fe(OH)_3_(s); Mn^2+^ + 2OH^–^⇋ Mn(OH)_2_(s) (1)

Fe(OH)_3_(s) + (*n*-3)OH^–^⇋ Fe(OH)*_n_*^3-*n*^; Mn(OH)_2_(s) + (*n*-2)OH^–^⇋ Mn(OH)*_n_*^2-*n*^ (2)

Mn(OH)*_n_*^2-*n*^ + Fe(OH)*_n_*^3-*n*^ → MnFe_2_O_4_ + H_2_O (3)

**Figure 4:**
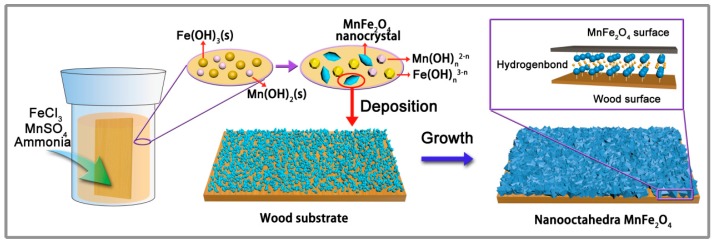
Possible schematic illustration of the preparation of the MW.

On the basis of the results mentioned above, a schematic illustration of the creation of nanooctahedra MnFe_2_O_4_ on the wood surface through a low temperature hydrothermal process is proposed in Figure 4. The precursors (FeCl_3_·6H_2_O and MnSO_4_·H_2_O) were dissolved into the deionized water and provided Mn^2+^ and Fe^3+^ ions at first. After adding ammonia solution, the Mn^2+^ and Fe^3+^ ions with the hydroxyl ions in the solution transformed into Mn hydroxides and Fe hydroxides, respectively. The Fe and Mn hydroxides dissolved in the mixed solution because of the presence of high concentrations of the ammonia and converted to the Mn(OH)*_n_*^2-*n*^ and Fe(OH)*_n_*^3-*n*^ with massive hydroxyl groups. Then, the Mn(OH)*_n_*^2-*n*^ and Fe(OH)*_n_*^3-*n*^, as the growth unit, formed the MnFe_2_O_4_ nanocrystal nucleus by the dehydration reaction. Additionally, the plentiful hydroxyl groups of the wood surface absorbed the MnFe_2_O_4_ nanocrystal for its the OH-rich surface that could be due to the high surface activity and large surface-to-volume atomic ratio on the MnFe_2_O_4_ nanoparticle’s surface. Finally, with the hydrothermal advanced, the MnFe_2_O_4_ nanocrystal developed a more stable MnFe_2_O_4_ layer on the wood surface and formed the MnFe_2_O_4_/wood composite.

**Figure 5:**
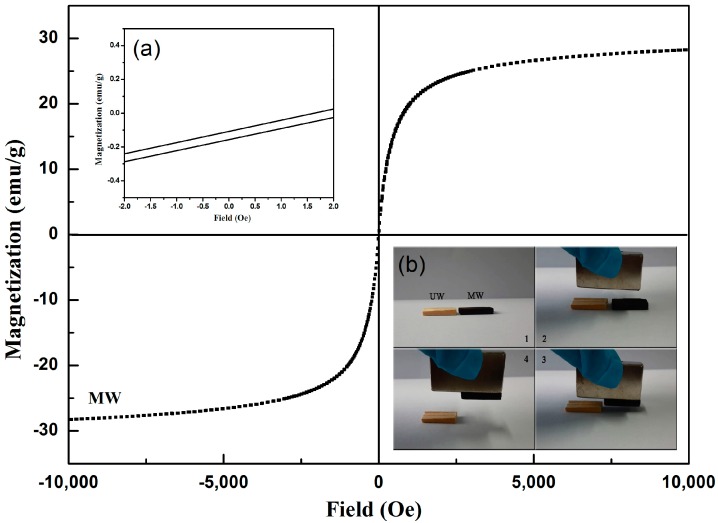
Magnetization of the MW as a function of the applied magnetic field. The inset (**a**) shows a magnification of one segment of the MW magnetization-field curves, and the inset (**b**) shows that the UW and MW samples were attracted by a magnet.

Figure 5 shows the magnetization-field curves of MW at 298 K. The saturation magnetization of MW was 28.24 emu/g, and it exhibited small hysteresis loops and low coercivity (<±5 Oe), as shown in the inset (a). Therefore, the MW showed a soft magnetism behavior [37,38,39]. The lower right corner illustration shows the camera images of the magnet attracting samples. In the inset (b), the UW lacks the response by a magnet, and the MW could be easily removed from the desktop by a magnet. These results revealed that the MW is an excellent soft magnetic material with sensitive magnetic response [40].

**Figure 6:**
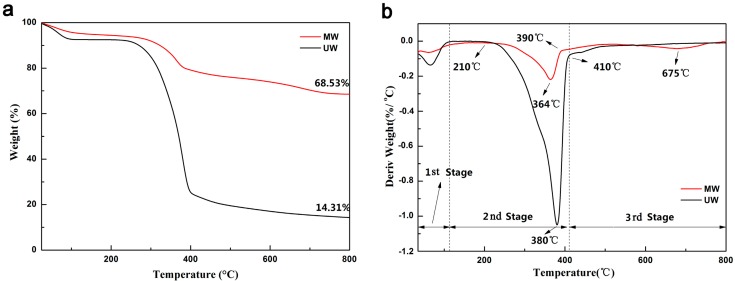
(**a**) Thermogravimetric (TG) and (**b**) differential thermogravimetric (DTG) curves of the UW and the MW under a nitrogen atmosphere.

The thermal stability of MW and UW was determined by TG and DTG under nitrogen atmosphere as shown in Figure 6. In the first stage (20 °C–110 °C), a little weight loss (UW of 7.43%; MW of 4.52%) was observed, which was related to the release of moisture from the samples. Thus, the water content of the UW and MW was 7.43% and 4.52%, respectively. In the second stage (110–410 °C), the weight/mass-loss rates of UW and MW were approximately 68.37% and 16.80%, respectively. The main weight loss was mainly attributed to the pyrogenic decomposition of hemicellulose and cellulose. The pyrolysis maximum rates of UW and MW occurred at 364 and 380 °C, respectively. Furthermore, the maximum rate of UW of −0.2189%/°C was higher than MW −1.049%/°C on the DTG curves, which could be caused by the higher content of nanooctahedra MnFe_2_O_4_ on the samples. In the third stage (410–800 °C), the weight loss of the MW reaches, 10.15% and the maximum pyrolysis rate occurred at 570 °C in which the nanooctahedra MnFe_2_O_4_ protects and improves the pyrolysis temperature of the wood. Finally, the MW still had larger residues (68.53%) with carbon residues and MnFe_2_O_4_, while the remainder of the UW with carbon residues was only 14.31% at 800 °C. This phenomenon indicated that the nanooctahedra MnFe_2_O_4_ deposited on the wood surface and had a large proportion in the MW. In summary, the thermal stability of the wood had been improved after the hydrothermal reaction and the existence of the potential in the fire-resistant performance.

**Figure 7:**
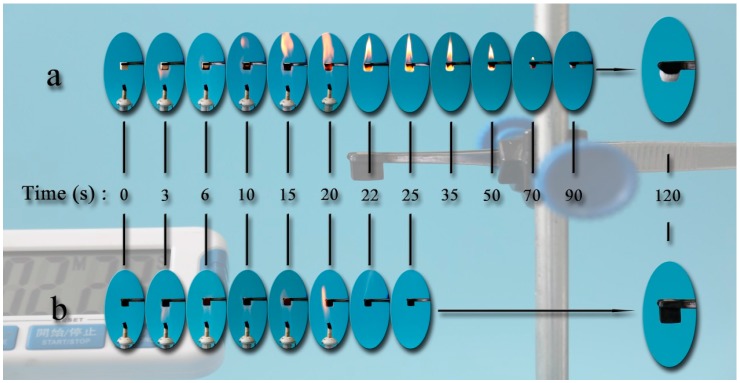
Fire-resistant properties of (**a**) the UW and (**b**) the MW.

In order to test the fire-resistant property, it was of great significance to test the ignitability of the UW and MW on an alcohol lamp flame. Figure 7 shows the digital photos of the UW and the MW on the alcohol lamp flame for the first 20 s. In Figure 7a, the UW caught on fire at 6 s and was incinerated to ash after 70 s. After removing the alcohol lamp at 20 s, the flames still burned strongly until 35 s. After 35 s, the flame had been gradually extinguished, and it became ash. By contrast, the MW could not be lit by the alcohol lamp burning for the first 20 s in Figure 7b. After removing the alcohol lamp, the shape and volume of the MW had no apparent change. These results indicated that the MnFe_2_O_4_/wood composite was more suitable than the untreated wood and might be applied in building and special decoration materials. This enhanced fire resistance could be ascribed to the special construction of the sample by which the dense MnFe_2_O_4_ layer formed over the cell wall on the wood surface prevented oxidation and heat transfer from proceeding into the inner portion of the wood cell walls, which could be observed from the scanning electron microscope. 

**Figure 8:**
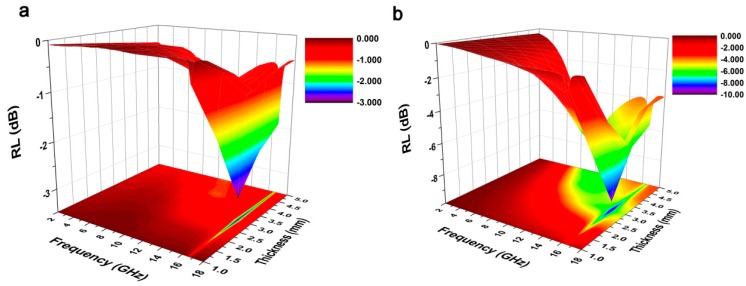
Frequency dependence of the reflection loss (RL) for (**a**) the UW and (**b**) the MW by three-dimensional and color-filling patterns in the frequency range of 2–18 GHz.

To investigate the electromagnetic absorption property of the UW and the MW, the reflection loss (RL) values were calculated by the transmission line theory [41].

(4) $$Z_{in}=Z_{0}\sqrt{\mu_{r}/\varepsilon_{r}}\tanh\left[j\left(\frac{2\pi fd}{c}\right)\sqrt{\mu_{r}\varepsilon_{r}}\right]$$

(5) $$RL\left(dB\right)=20\log\left|\frac{Z_{in}-Z_{0}}{Z_{in}+Z_{0}}\right|$$

where *Z_in_* is the input impedance of the samples, *Z*_0_ is the impedance of air, $\mu_{r}$ is the complex permeability,$\varepsilon_{r}$ is the complex permittivity, *d* is the thickness of the samples, *f* is the frequency of microwaves and *c* is the velocity of light. Figure 8 shows RL curves of the UW and MW by three-dimensional and color-filling patterns in the frequency range of 2–18 GHz. In Figure 8a, the minimum RL values of the UW reached −3.0 dB at 16.64 GHz, and the RL values of the UW were close to zero at another frequency, which indicated that the main loss mechanism of the UW was only the resonance of the wood at a certain frequency at 16.64 GHz. By contrast, the MW reached −9.3 dB at 16.48 GHz with a thickness of 3 mm in Figure 8b. Moreover, it could be observed that both the minimum reflection loss value and the absorption bandwidth of the MnFe_2_O_4_/wood composite had improved greatly compared with the untreated wood, indicating that the electromagnetic absorption property of the MnFe_2_O_4_/wood composite had been enhanced through the growth of the MnFe_2_O_4_ on the wood. Thus, it might provide a potential application in the field of indoor electromagnetic wave absorbers.

## 4. Conclusions

In summary, we had successfully synthesized the MnFe_2_O_4_/wood composite via a low-temperature hydrothermal method. The nanooctahedra MnFe_2_O_4_ had been tightly located on the wood surface through the hydrogen bonding interactions. The MnFe_2_O_4_/wood composite expressed superior performance of soft magnetism, fire resistance and electromagnetic wave absorption, which could be applied to some fields for special decoration and indoor electromagnetic wave absorption.

## Figures and Tables

**Table 1:** Surface composition of the UW and the MW.

UW		MW		Assignment
The excited electron O1s	Binding energy (eV)	The excited electron O1s	Binding energy (eV)	
O1	533.18	O3	533.15	C–O
O2	531.83	O4	531.55	O–H, C=O
		O5	530.23	Mn–O, Fe–O
